# Chile Pepper (*Capsicum*) Breeding and Improvement in the “Multi-Omics” Era

**DOI:** 10.3389/fpls.2022.879182

**Published:** 2022-05-03

**Authors:** Dennis N. Lozada, Paul W. Bosland, Derek W. Barchenger, Mahdi Haghshenas-Jaryani, Soumaila Sanogo, Stephanie Walker

**Affiliations:** ^1^Department of Plant and Environmental Sciences, New Mexico State University, Las Cruces, NM, United States; ^2^Chile Pepper Institute, New Mexico State University, Las Cruces, NM, United States; ^3^World Vegetable Center, Tainan, Taiwan; ^4^Department of Mechanical and Aerospace Engineering, New Mexico State University, Las Cruces, NM, United States; ^5^Department of Entomology, Plant Pathology and Weed Science, New Mexico State University, Las Cruces, NM, United States; ^6^Department of Extension Plant Sciences, New Mexico State University, Las Cruces, NM, United States

**Keywords:** genome-wide association study, genomic selection, heat profile, high-throughput phenotyping, *Phytophthora capsici* resistance, single nucleotide polymorphisms, speed breeding, yield

## Abstract

Chile pepper (*Capsicum* spp.) is a major culinary, medicinal, and economic crop in most areas of the world. For more than hundreds of years, chile peppers have “defined” the state of New Mexico, USA. The official state question, “*Red or Green*?” refers to the preference for either red or the green stage of chile pepper, respectively, reflects the value of these important commodities. The presence of major diseases, low yields, decreased acreages, and costs associated with manual labor limit production in all growing regions of the world. The New Mexico State University (NMSU) Chile Pepper Breeding Program continues to serve as a key player in the development of improved chile pepper varieties for growers and in discoveries that assist plant breeders worldwide. Among the traits of interest for genetic improvement include yield, disease resistance, flavor, and mechanical harvestability. While progress has been made, the use of conventional breeding approaches has yet to fully address producer and consumer demand for these traits in available cultivars. Recent developments in “multi-omics,” that is, the simultaneous application of multiple omics approaches to study biological systems, have allowed the genetic dissection of important phenotypes. Given the current needs and production constraints, and the availability of multi-omics tools, it would be relevant to examine the application of these approaches in chile pepper breeding and improvement. In this review, we summarize the major developments in chile pepper breeding and present novel tools that can be implemented to facilitate genetic improvement. In the future, chile pepper improvement is anticipated to be more data and multi-omics driven as more advanced genetics, breeding, and phenotyping tools are developed.

## Introduction

Chile pepper (*Capsicum* spp.) is an important vegetable crop in most regions of the world, including the state of New Mexico, in the Southwestern USA, where chile pepper is regarded as a major agricultural commodity. The diverse utilization of chile peppers from culinary (as a major spice), to health, and industrial uses has driven its cultivation worldwide. Total global production was ~40 M tons in 2020, with the majority (36.1 M tons; 90%) comprised of fresh chile pepper (Food and Agriculture Organization of the United Nations, 2021). Top chile producers (in M tons) for the fresh market were China (16.7), Mexico (2.8), Indonesia (2.7), Turkey (2.6), and Spain (1.5). India was the highest producer of dry chile pepper with 1.7 M tons, followed by Thailand (0.32), China (0.31), Ethiopia (0.30), and Bangladesh (0.16; Food and Agriculture Organization of the United Nations, 2021). In terms of consumption, Vietnam, India, and United States were the top consumers in 2018 with consumption (in kilotons) of 166, 86, and 68 (World Pepper Market Report, 2019). In the United States, the *per capita* fresh chile pepper consumption has more than doubled in the last 40 years, whereas the *per capita* consumption of dried chile peppers has increased by more than 46% (Lillywhite and Tso, 2021).

While the total global production for both dry and fresh chile pepper has been generally increasing (Food and Agriculture Organization of the United Nations, 2021), this might not be sufficient to cope with the growing demand for chile peppers and the increasing world population that is projected to be 10 billion by 2050 (Hickey et al., 2019; Pandey et al., 2021). The total production in New Mexico has been dynamic over the past three decades, with an overall decreasing trend in recent years (USDA NASS, 2020); this is a consequence, among others, of the decreasing acreages brought about by the unavailability of labor and costs associated with manual harvesting, even as consumption grows (Walker and Funk, 2014; Joukhadar et al., 2018). Incorporating different multi-omics approaches, such as genomics-assisted breeding, transcriptomics, epigenomics, and metabolomics, among others, in dissecting the genetic basis of traits for chile pepper breeding and improvement can aid in advancing solutions to these issues. Recent advances in molecular genetics and genomics, high-throughput phenotyping, development of efficient machine harvesters, and integrating robotics with agriculture (i.e., “agricultural robotics”), can be applied to chile pepper breeding to mitigate the effects of the various issues facing production. It would therefore be important to discuss these omics approaches in the context of chile pepper breeding and improvement.

In this review, the different omics tools that plant breeders can utilize to address constraints in chile pepper production are discussed. First, the relevance of maintaining genetic diversity in germplasm collections is highlighted, followed by the exploration of the different tools currently being used for the genetic improvement of chile pepper. Specifically, the various “omics” approaches that have been implemented for the genetic improvement of several target traits for chile pepper breeding including yield, resistance to *Phytophthora capsici,* heat levels, and mechanical harvestability are described. The role of robotics approaches to aid in mechanical harvesting of chile pepper is also discussed. This review serves as valuable resource for chile pepper breeders in developing a path forward, creating breeding pipelines, and establishing collaborations for the genetic improvement of this critically important crop.

## Gene Bank Collections and Genetic Diversity

It is essential to maintain genetic diversity in breeding programs for the introduction of different beneficial alleles for the improvement of current cultivars. Without diversity, no progress can be made in a breeding program. Gene banks and germplasm collections are valuable repositories of seeds, the genetic variation of which can be used for genome-wide improvement (Tanksley and McCouch, 1997; Singh et al., 2019). Profiling DNA sequences using molecular markers, such as single nucleotide polymorphism (SNP) derived from genotyping-by-sequencing (GBS), can reveal genetic differences among accessions, which, in turn, can reflect the level of genetic diversity in current germplasm. Most of the previous genetic diversity studies in chile pepper were conducted using populations originating from certain geographic areas of the world including Spain (Pereira-Dias et al., 2019), Ethiopia (Solomon et al., 2019), Mexico (Taitano et al., 2018), and a global collection with representative accessions from Europe, Asia, Africa, and the Americas (Taranto et al., 2016); therefore, there is a need to assess the genetic diversity of existing chile pepper germplasm from other growing regions. For New Mexican germplasm, the genetic diversity of chile pepper landraces from Northern New Mexico was evaluated using random amplified polymorphic DNA (Votava et al., 2005) and it was observed that the Northern New Mexico cultivars were more closely related to the Mexican varieties than the Southern New Mexican cultivars. Simple sequence repeat (SSR) markers characterized the genetic diversity of 147 *C. frutescens* accessions from 25 countries and clustered the population into seven major groups consistent with their geographic origins (Zhong et al., 2021). In another study, SSR marker-based genetic diversity of 32 *Phytophthora capsici* resistant accessions of *C. annuum* from Ethiopia and India were evaluated and the genotypes clustered into three genetic resistance groups (Rabuma et al., 2020). Recently, genotyping using 66,000 GBS-derived SNP markers demonstrated that genetic structure was related to fruit (pod) architecture and morphology across the different species (Lozada et al., 2021a). Nevertheless, genetic diversity was relatively low; hence, there is a need to introduce new beneficial alleles to increase genetic diversity in current New Mexican chile pepper germplasm by introducing genes from other breeding programs and from wild relatives or domesticated species. Genotyping of the NMSU New Mexican pod-type cultivars using GBS-derived SNP markers revealed remarkable genetic differences across genome-wide SNP sites. For example, the “NuMex Heritage Big Jim” (Bosland and Coon, 2013) and “NuMex Sandia Select” (Bosland and Coon, 2014) derived from the heirlooms “NuMex Big Jim” (Nakayama, 1975) and “NuMex Sandia” (Harper, 1950) respectively, formed separate clusters with their parental lines. This could be a consequence of extensive and multiple cycles of breeding and phenotypic single-plant selections in the breeding program (Lozada et al., 2021a). Genetic improvement of current germplasm, then, can be linked to the differences and variation in the alleles and genes present that were selected on each cycle of phenotypic selection in the breeding program.

The NMSU Chile Pepper Breeding and Genetics program houses the New Mexico Capsicum Accessions (NMCA), a collection of more than 2,100 accessions from 22 different species, including the five domesticated species—annuum, baccatum, chinense, frutescens, and pubescens, originating from various geographic regions of the world. Established in 1985, the NMCA collection is regarded as one of the most diverse germplasm banks in terms of the number of species for the chile pepper (Barchenger and Khoury, in press). To discover the true potential of the seeds stored in gene banks, it is necessary to not only examine the phenotypes for traits of interest (e.g., yield, disease resistance), but also to survey the favorable alleles present in these collections. To date, less than 10% of the NMCA accessions have been genotyped using genome-wide SNP markers; hence most of the lines that could possess beneficial genes remain untapped for cultivar development and genetic improvement. The landraces, for instance, have a high capacity as sources of resistance to local biotic and abiotic stresses and could serve as a reservoir of genetic variability for the plant breeder (Zeven, 1998; Votava et al., 2005). These landraces are the earliest form of cultivars and may contain more genetic variation than the modern domesticated varieties that were selected for optimal performance (McCouch, 2004). It is thus necessary for the alleles from the accessions stored in gene banks to be transferred to cultivar development programs (Dreisigacker et al., 2021). In the future, it would be important to profile the DNA sequences of the NMCA accessions, including the landraces from Northern New Mexico, using high-density genome-wide markers, such as SNPs, to mine for favorable alleles that control important horticultural traits for introduction into adapted local varieties.

A concerted effort for the preservation of the chile pepper across the different growing areas of the world through the Global Capsicum Conservation Strategy, which NMSU is a partner, is currently ongoing, and this would open opportunities for germplasm exchange, protection, maintenance, and characterization using novel molecular marker platforms (Barchenger and Khoury, *in press*). Previously, the distribution and conservation status of wild relatives of chile peppers were evaluated using *ex* and *in situ* assessments and 18 out of the 37 (48%) of the wild taxa examined were categorized as “high priority” for further conservation (Khoury et al., 2020). The cost of high-throughput genotyping is continuously decreasing (Torkamaneh et al., 2018; Ayalew et al., 2019), and this can ultimately leverage genomics-assisted breeding to accelerate genetic improvement for current chile pepper cultivars. Additionally, profiling of gene bank accessions using high-density genome-wide SNP markers and ultimately integrating this information with passport data could provide models for the distribution, routes of evolution, and domestication of chile pepper (Tripodi et al., 2021), giving further insights for potential improvement and breeding strategies. In addition to the germplasm stored at NMCA, the US Department of Agriculture Germplasm Research Information Network (USDA GRIN) houses at least 500 accessions belonging to nine species of *Capsicum* from more than 50 different growing areas including Brazil, China, Colombia, Iran, Greece, Mexico, and United States (USDA GRIN, 2022). The genomic richness and diversity of germplasm stored in chile pepper gene banks remains an indispensable resource that breeders should utilize for future cultivar improvement and development.

## Genome-Wide Mapping and QTL Identification for Chile Pepper Breeding and Improvement

**Figure 1:**
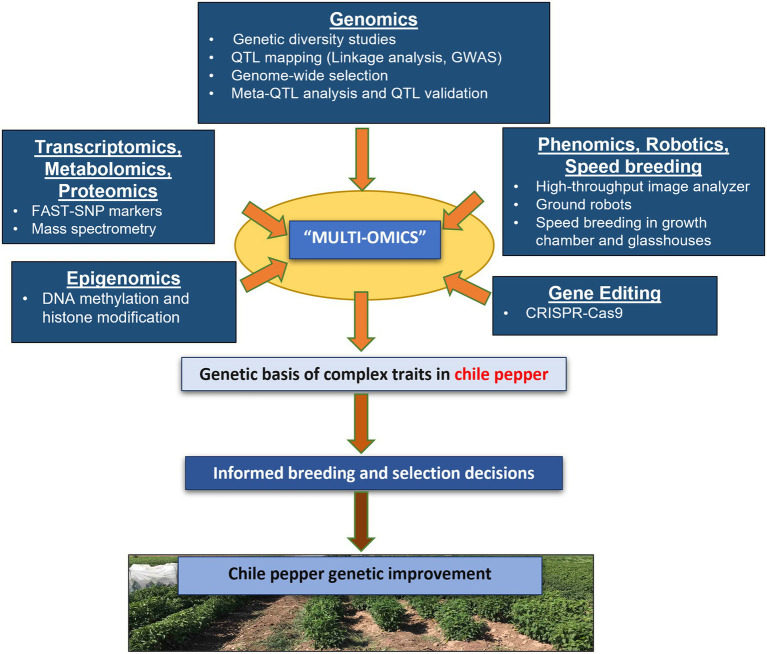
Harnessing the power of multi-omics approaches for genetic improvement in chile pepper. Genomics-assisted breeding approaches, including genetic mapping (GWAS and QTL analysis) and genomic selection can facilitate the dissection of the genetic basis of complex traits in chile pepper by identifying genomic regions associated with important traits. Transcriptomics and epigenomics can render further insights into the expression and regulation of expression of these genetic systems. Gene editing (e.g., using CRISPR-Cas9) allows a precise and accurate modification of genes using a guide RNA (gRNA) for a targeted alteration of the genomic sequence, whereas phenomics approaches can expedite trait collection in the field. Speed breeding can accelerate development to increase genetic gain. Breeders can ultimately use the information derived from these various omics tools either exclusively or in combination with other approaches to select for and develop improved cultivars of chile pepper.

The availability of whole-genome sequences for chile pepper (Kim et al., 2014; Qin et al., 2014; Hulse-Kemp et al., 2018) has facilitated molecular breeding, marker development, and marker-assisted selection for chile pepper genetic improvement. Modern technologies have driven genetic improvement of important traits for horticultural crops through multi-omics approaches arising from information derived from the whole-genome sequence (Hao et al., 2020). The genetic architecture of important complex traits in chile pepper can be examined using different genetic mapping strategies such as linkage analysis and genome-wide association studies (GWAS; Figure 1). These mapping approaches identify regions in the genome called quantitative trait loci (QTL) that affect variation of the ultimate phenotype. The main difference is the control over recombination: there is a higher rate of recombination in GWAS as a result of using diverse, natural populations compared to linkage mapping, hence rendering a higher mapping resolution (Zhu et al., 2008; Myles et al., 2009). As an approach, GWAS allows the discovery of multiple alleles and is flexible and fast, as there is no need to develop inbred lines from biparental crosses (Lander and Schork, 1994).

Genotyping-by-sequencing (GBS) has been developed and implemented as a robust method to discover single nucleotide polymorphisms (SNP) for various downstream applications (Elshire et al., 2011). The flexibility, cost-effectiveness, and efficiency of a GBS platform makes it one of the main next-generation sequencing (NGS)-based approaches for SNP marker discovery for genomic diversity studies, genetic linkage analysis, GWAS, and genomic selection (He et al., 2014b). In chile pepper, GWAS has been implemented to examine the genetic basis of various traits including capsaicinoid content, response to infection by *Phytophthora capsici*, and various fruit parameters, such as fruit weight, length, and width (Table 1). Genome-wide mapping approaches using GBS-derived SNP markers have dissected the genetic architecture of important horticultural traits in the NMSU Chile Pepper Breeding Program. The information derived from GWAS can direct breeding and selection decisions using molecular marker-based platforms for the genetic improvement of chile pepper.

**Table 1:** Summary of major genome-wide association studies (GWAS) conducted for different traits in chile pepper.

Trait	No. of individuals	Species	Marker type^a^	GWAS models^b^	Total no. of markers	No. of significant marker–trait associations	Chromosomes	References
Capsaicinoid content	208	*Capsicum annuum, Capsicum chinense, Capsicum frutescens*	GBS-SNP	CMLM	109,610	99	1, 3, 6, 10, 11^c^	Han et al., 2018
Capsaicinoid content	96	*C. annuum*	SSR	GLM and MLM	176	5	1	Nimmakayala et al., 2014
Capsaicinoid content	94	*C. annuum*	GBS-SNP	MLM (EMMAX)	7,331	86	1, 2, 3, 5, 6, 9, 10, 11	Nimmakayala et al., 2016
Fruit length	230	*C. annuum*	GBS-SNP	CMLM	187,966	8	3, 4, 5, 7, 11	Lee et al., 2020
Fruit position	230	*C. annuum*	GBS-SNP	CMLM	187,966	52	3, 5, 12	Lee et al., 2020
Fruit shape	220	*C. annuum*	GBS-SNP	LMM (GEMMA)	746,000	8	3, 10, 11	Colonna et al., 2019
Fruit shape	2,059	*C. annuum*	GBS-SNP	MLM (GEMMA)	26,566	6^d^	10, 11	Tripodi et al., 2021
Fruit weight	96	*C. annuum*	SSR	GLM and MLM	176	11	1, 2, 4, 5, 8, 9, 10	Nimmakayala et al., 2014
Fruit weight	94	*C. annuum*	GBS-SNP	MLM (EMMAX)	7,331	61	1, 2, 3, 4, 5, 6, 8, 9, 10, 11, 12	Nimmakayala et al., 2016
Fruit weight	230	*C. annuum*	GBS-SNP	CMLM	187,966	101	1, 2, 4, 6, 7, 8, 9, 10, 11, 12	Lee et al., 2020
Fruit width	287	*C. annuum, Capsicum baccatum, C. chinense, C. frutescens*	SLAF-SNP	FaST-LMM	594,429	3	1, 8, 12	Wu et al., 2019
Fruit width	230	*C. annuum*	GBS-SNP	CMLM	187,966	281	7, 9, 12	Lee et al., 2020
Number of flowers per axil	287	*C. annuum, C. baccatum, C. chinense, C. frutescens*	SLAF-SNP	MLM (EMMAX)	594,429	12	1, 4, 5, 6, 7, 9, 10, 11, 12	Wu et al., 2019
Number of pedicels per axil	2,059	*C. annuum*	GBS-SNP	MLM (GEMMA)	26,566	4^d^	6	Tripodi et al., 2021
Pedicel position at anthesis	2,059	*C. annuum*	GBS-SNP	MLM (GEMMA)	26,566	6^d^	2, 12	Tripodi et al., 2021
Pericarp thickness	287	*C. annuum, C. baccatum, C. chinense, C. frutescens*	SLAF-SNP	FaST-LMM	594,429	4	1, 8, 11, 12	Wu et al., 2019
Pericarp thickness	230	*C. annuum*	GBS-SNP	CMLM	187,966	9	4, 6, 7, 11, 12	Lee et al., 2020
*Phytophthora capsici* resistance	352	*C. annuum*	GBS-SNP	CMLM	507,713	117	5, 7, 11^c^	Siddique et al., 2019

a *GBS-SNP*, Genotyping-by-sequencing (GBS)-derived single nucleotide polymorphism (SNP) markers; *SSR*, Simple sequence repeats; SLAF-SNP, Specific locus amplified fragment SNP.

b *CMLM*, Compressed mixed linear model; *FaST*-*LMM*, Factored spectrally transformed linear mixed model; *EMMA*, Efficient mixed model association; *EMMAX*, Efficient mixed model (expedited); *GEMMA*, Genome-wide efficient mixed model analysis; *GLM*, Generalized linear model; *MLM*, Mixed linear model.

c Co-localized with QTL identified from linkage mapping.

d Marker–trait associations with *p* < 1.00E-09.

The identification of disease resistance QTLs across different chile pepper germplasm can accelerate breeding for improved resistance. Chile pepper root rot caused by the oomycete *Phytophthora capsici* remains as one of the most destructive diseases affecting *Capsicum* production globally, a century after it was first described in New Mexico by Leonian (1922). While management practices can help mitigate the effects of *P. capsici* on chile pepper production (Sanogo and Ji, 2012), breeding and selection for disease-resistant cultivars are still the most cost-effective approach (Xu et al., 2016; Siddique et al., 2019). The short arm of chromosome P5 is a major genetic hotspot containing QTL for *P. capsici* resistance (Mallard et al., 2013; Rehrig et al., 2014; Siddique et al., 2019; Du et al., 2021; Lozada et al., 2021b,c), and this information facilitates breeding efforts for resistance to *P. capsici*. A set of New Mexico recombinant inbred lines (NMRIL) derived from the hybridization between the resistant landrace, CM-334, and the susceptible cultivar “Early Jalapeno” was previously developed at NMSU to facilitate a better understanding of the complex inheritance of *P. capsici* root rot resistance and the characterization of different races for breeding resistant cultivars (Sy et al., 2008). Recently, large main effect and QTL interaction in chromosomes P5 and P8 that can be used as a basis for marker-assisted selection were identified using the NMRIL (Lozada et al., 2021b). Genetic mapping further mapped SNP loci related to other diseases in chile pepper, such as anthracnose on chromosomes 2 and 4, where three major QTLs for resistance were located between two SNP markers within 17 cM distance in chromosome 4 (Mahasuk et al., 2016). Powdery mildew resistance QTLs on chromosome 4 were previously mapped using a BC_1_F_2_ (Kim et al., 2017) and F_2_ and F_2:3_ mapping populations identifying a major locus, *PMR1*, for resistance to powdery mildew (Jo et al., 2017). Loci associated with bacterial wilt and root knot nematode have been identified in chromosomes 10 (Du et al., 2019) and 9 (Changkwian et al., 2019), respectively. QTLs related to viral diseases including cucumber mosaic virus (Choi et al., 2018; Li et al., 2018b), pepper mottle virus (Holdsworth and Mazourek, 2015; Venkatesh et al., 2018), and pepper mild mottle virus (Yang et al., 2012), among others, have also been mapped across various chromosomes in the chile pepper genome. The large effect QTLs identified for various diseases in chile pepper can be confirmed and validated using different breeding populations for application in breeding programs.

Chile peppers are good sources of vital nutrients, such as capsaicinoids, vitamins A, C, and folate, which can also be improved using genome-wide mapping approaches. An improved nutritional content that can place an “added quality value” to chile pepper, could potentially increase consumer consumption and acceptance. “NuMex LotaLutein,” a serrano type with improved lutein content, a compound necessary for healthy eyesight, has been released recently (Guzmán et al., 2020). A survey of vitamin content among a diverse set of chile peppers showed eight different genotypes to have higher vitamin A concentration than sweet potato (*Ipomea batatas*), and a total of 16 genotypes had higher vitamin C content than kiwi (*Actinidia arguta*; Kantar et al., 2016), demonstrating that chile peppers can be good alternative sources of important minerals to combat nutrient deficiencies. Previous analyses have focused on the identification of QTL related to carotenoid and capsaicinoid content, whereas mapping for loci associated with nutritional value (provitamin A, vitamin C, etc.) using linkage and GWAS has yet to be conducted in chile pepper. The *C. annuum phytoene synthase 1* (*CaPSY1*) gene has been identified as a major locus associated with carotenoid metabolism at maturity (Wei et al., 2020), and can be a potential target for molecular breeding to increase carotenoid content. Linkage mapping has identified several genomic regions and candidate genes related to the development of capsaicinoids on chromosomes 1, 2, 3, 4, and 10 (Han et al., 2018). Using a whole-genome sequencing-based QTL sequencing strategy, a major QTL on chromosome P6 was identified for capsaicinoid biosynthesis in the pericarp of *C. chinense* (Park et al., 2019). Other genomic regions related to capsaicinoid content were mapped in chromosomes 3 (Lee et al., 2016), 4 and 7 (Ben-Chaim et al., 2006), demonstrating the genetic complexity of capsaicinoid content. These regions can therefore be targeted for molecular breeding and selection using genomics-assisted approaches. An F_2_ mapping population mapped the *Pun1* gene which encodes a putative acyltransferase for capsaicinoid biosynthesis on the short arm of chromosome 2 (Stewart Jr et al., 2005). As the genetic basis for nutritional quality traits, primarily the capsaicinoids, have only been examined primarily by biparental QTL mapping using low-density markers and low-throughput phenotypic data, it would be necessary to employ higher resolution GWAS scans to complement linkage mapping in dissecting the genetics of these traits in chile pepper.

Once the significant loci are identified using association mapping, molecular markers can be developed and used routinely in the breeding program for marker-assisted breeding and selection (Lozada et al., 2018; Ren et al., 2019; Ma et al., 2020). For example, the KBioscience Competitive Allele Specific assays (KASP^®^; He et al., 2014a; Semagn et al., 2014), can be developed based on the flanking sequences of significant markers identified from GWAS. As an approach, the single-plex fluorescence resonance energy transfer (FRET)-based KASP system is cost-effective, high-throughput, and flexible (Jatayev et al., 2017; Majeed et al., 2019). Currently, in chile pepper, the application of KASP has been limited as these marker assays have only been developed and applied for marker-assisted selection for resistance to diseases, such as bacterial leaf spot caused by the bacterium *Xanthomonas euvesicatoria* (Holdsworth and Mazourek, 2015) and of the fertility restorer genes (Zhang et al., 2021). Given this boundless potential for genetic improvement through molecular breeding as demonstrated on its previous applications in other crops, including chile pepper to a certain extent, the KASP genotyping platform could be integrated in chile pepper breeding pipelines to facilitate the selection of desirable alleles for important traits. KASP assays for the *MAP1* marker on chromosome 2 (Rodríguez-Maza et al., 2012) and markers flanking *P. capsici* resistance QTLs on the short arm of chromosome 5 can be developed for screening heat levels and disease resistance, respectively. SSR markers previously developed and identified in other chile pepper breeding populations can be further converted to KASP for validation and marker-assisted selection of disease-resistant lines (Table 2). Information from genetic mapping could be integrated with genome-wide selection and phenomics-aided approaches in breeding programs to select for complex traits in chile pepper breeding programs.

**Table 2:** Simple sequence repeat markers for conversion to allele-specific KASP assays and validation using for marker-assisted breeding of *Phytophthora capsici* resistance.

Marker Name	Primer sequences	Chr.	Position (Mb or cM)	Reference
P217-220-3	F: GAGTAAACCGATAATCCAAT	10	217.48^M^	Xu et al., 2016
	R: ATGTTAGTTAGGAGGAATTA			
P217-220-4	F: TTCCTTTATGTCTAGGCTTT		217.51	
	R: CAGTTTTCAGGTACATTACT			
P220-229-54	F: TAATGGGGTTCAACATCTAC		228.31	
	R: CTTTTTGTTCCTTATCACTT			
P52-11-21	F: CAATCCAAACAAGTCCTAAG		229.19	
	R: GGTGCAATTGAAAATCTAAG			
P52-11-41	F: TTGATGAGATGGGAAGTAAA		231.75	
	R: CACCAACAATAATAGAACTACA			
P230-233-11	F: ATAGAATGACTTCCAGGCAA		232.06	
	R:AAAGGTAAGGAGTAAGGCTG			
CAMS089	F: AACAGCGCTGATCCTTTACC	3	0^c^	Minamiyama et al., 2007
	R: CAACATCACAGTGGCAGAAGA			
CAMS865	F:AGAAATCGTGGTTGGGTGAG		37.6	
	R: CACTTTGGCACATTTTGCTG			
HPMS1-139	F: CCAACAGTAGGACCCGAAAATCC		42	
	R: ATGAAGGCTACTGCTGCGATCC			
Hpms1-1	F: AACCCAATCCCCTTATCCAC	1	73–101^c^	Lee et al., 2004
	R: GCATTAGCAGAAGCCATTTG			
Hpms1-117	F: CGCATATACATACATAAATTCTTTC	1	109–129	
	R: TCAACATCTCACCGAAGCTG			
Hpms2-2	F: ATCTTCTTCTCATTTCTCCCTTC	11	195–206	
	R: TGCTCAGCATTAACGACGTC			
CAeMS-068	F: ATCAAATCTCAACACATGGTGGCT	5	12.46–12.49^M^	Wang et al., 2016
	R: GTTTACTGTATCTCCGGCCCTGTCA			
ZL6203	F: AGGTGGTACAAACTTCCTATG		25.8801–25.8802	
	R: GGGAGCTCTGTTCTTTATGTA			
ZL6726	F: TCCAGCCATCCATTATTTCAT			
	R: ATCCCGAACTGCCAATAATTA		29.09721–29.09736	
ZL7825	F: CTTTTGGTGAGATGTGTGTTT		33.29099–33.29114	
	R: ACCCCCTACTCCCTTTTTATA			

M In mega base pairs (Mb);

c In centimorgans (cM).

## GEBV-Based Breeding in Chile pepper

The limitation of GWAS is that it may not identify loci of small effects (i.e., the case of missing heritability; Korte and Farlow, 2013). Another marker-assisted selection approach, genomic selection (GS), uses genome-wide marker information to predict the genomic estimated breeding values (GEBV) of individuals (Meuwissen et al., 2001) and can be used to complement GWAS. In GS, a training population comprised of individuals with both genotype and phenotype data predicts the GEBV of selection candidates in the validation (test) population consisting of lines having only genotype data (Crossa et al., 2017). The correlation between the GEBV and the observed phenotypes is called prediction accuracy, *r*, and is affected by several factors, such as the size of the training population, number of markers, genetic relatedness between the training and test populations, and prediction models used (Spindel et al., 2015; Cericola et al., 2017; Norman et al., 2018; Larkin et al., 2019; Lozada et al., 2019).

Achieving optimal prediction accuracy is a key for increasing genetic gain, which is the change in phenotypic performance of populations, in plant breeding programs (Xu et al., 2020). Reducing the length of the breeding cycle for cultivar development is one of the advantages of GS-based breeding strategies compared to conventional phenotypic selection to increase the rate of genetic gain (Voss-Fels et al., 2019). While GS has been successfully applied to different traits across various crops, such as rice (*Oryza sativa* L.; Huang et al., 2019), bread wheat (*Triticum aestivum* L.; Belamkar et al., 2018; Juliana et al., 2018; Lozada and Carter, 2019), and soybean (Jarquin et al., 2016), these approaches still need to be implemented for the genomics-assisted breeding of chile pepper. To date, GS approaches have only predicted fruit-related traits in Korean chile pepper accessions, where high prediction accuracies *viz.* 0.73 (fruit shape), 0.75 (fruit length), 0.82 (pericarp thickness), 0.83 (fruit weight), and 0.84 (fruit width) were observed using Reproducing Kernel Hilbert space models (Hong et al., 2020). Currently, GS has not been applied in chile pepper improvement programs, specifically on the prediction of fruit yield and yield components, and hence, it is worthwhile to test these approaches in accelerating the genetic gain for these traits.

The breeding values, represented as GEBV, assist in selecting lines to advance in a breeding program and for selection of parents for hybridization. The GEBVs allow the prediction of individuals that will be “superior” and are suitable either as parental lines for hybridization or for next-generation advancement as the molecular marker profile of those individuals are similar to that of the training populations that have been observed to perform better in different field trials (Bhat et al., 2016). The GS approach predicts the performance of a hybrid allowing for a more effective utilization of genomic and financial resources in breeding programs (Dreisigacker et al., 2021). In general, the process of breeding, selection, and development of improved chile pepper varieties is long-term (~10 years), consisting of a series of single-plant selection schemes and replicated trials. As an approach, GS can be integrated in the chile pepper breeding program to accelerate the process of selection and release of improved chile pepper varieties through GEBV-based breeding and selection. The GEBV can be calculated by performing independent validations using a training population to predict the future performance of single-plant selections and replicated trials. Lines with high GEBV (and therefore, a better predicted performance) can be advanced in the breeding cycle to reduce time for cultivar release, thereby increasing gains achieved through selection. The training populations for GS in chile pepper breeding should be large enough to capture the optimal genetic relatedness between the training and test (validation) populations to achieve ideal prediction accuracies. Intraspecies GS (e.g., annuum predicting annuum) is also recommended to avoid the confounding effects of predictions across species and to maximize genetic relationships between the populations for improved selection accuracy.

## Phenomics and “Speed Breeding” to Accelerate Chile pepper Improvement

In plant breeding, phenotyping has lagged behind genotyping primarily due to the manual-based observation used for data collection and the number of lines that need to be evaluated in field trials (Shen et al., 2022). This “phenotyping bottleneck” (Furbank and Tester, 2011) is being addressed through the development of high-throughput, fast, and accurate methods in collecting phenotypic data. In chile pepper, the traditional approach of quantifying heat levels in fruit samples is based on an organoleptic test, where experienced raters score each sample. Developed by Wilbur Scoville, the test is known as the Scoville Heat Units (SHU; Scoville, 1912). This method, nonetheless, can be biased and subjective because of interpersonal differences. In addition, taster fatigue is a common phenomenon with the organoleptic method. Advanced analytical methods include the high-performance liquid chromatography (HPLC) that measures capsaicinoid levels as parts per million is then converted to SHU (Collins et al., 1995). Among the drawbacks for using HPLC in determining SHU levels include the relatively long turn-around time for the processing of samples and the costs associated with equipment operation and maintenance. Recently, efforts to develop cost-effective, fast, and direct detection of capsaicinoid content from samples using different techniques, such as voltammetry and near-infrared spectroscopy, have been made (Crapnell and Banks, 2021). For example, a graphene-based portable device that can be connected to a smartphone has been developed and high accuracy and collinearity between the SHU values derived from the machine and from spectroscopy were observed (Soleh et al., 2020). A colorimetric approach using the Gibbs reagent has also been employed for the rapid determination of capsaicinoid content and has a high correlation with HPLC (Ryu et al., 2017). Through using different HTP approaches, more lines can be evaluated in more locations to address the significant interactions between genotype and environment (G × E). This consequently would facilitate a better implementation of genomics-assisted selection for breeding heat levels in chile pepper.

**Figure 2:**
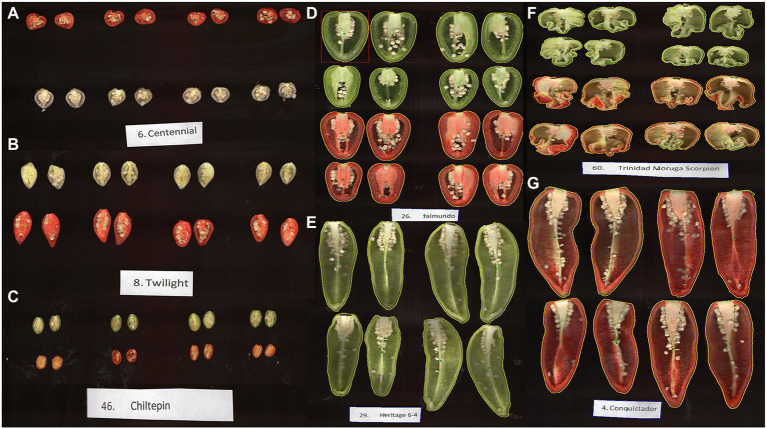
Phenotypic diversity among chile pepper evaluated for fruit morphology-related traits using the Tomato Analyzer v.4.0 program. **(A)** “NuMex Centennial” and **(B)** “NuMex Twilight” (*C. annuum*) are ornamental chile peppers. **(C)** Chiltepins (*Capsicum annuum var. glabriusculum*), commonly known as “bird peppers,” are regarded as the progenitors of the cultivated *C. annuum*. **(D)** “NuMex Jalmundo” (*C. annuum*) is a large-sized jalapeño. **(E)** “NuMex Heritage 6–4” and **(G)** “NuMex Conquistador” (*C. annuum*) are both New Mexican pod-type chile peppers. **(F)** “Trinidad Moruga Scorpion” (*C. chinense*) is a “superhot” chile pepper.

Performing indirect selection of primary traits can be implemented using highly heritable and correlated traits collected using HTP methods. Among the phenomics platform that has been developed is the Tomato Analyzer (TA), a morphometric and colorimetric tool for the phenotypic characterization of traits related to fruit architecture and morphology (Brewer et al., 2006; Gonzalo et al., 2009; Rodríguez et al., 2010). Initially developed for the phenotypic characterization of tomato (*Solanum lycopersicum*) fruit samples, the TA has been used extensively to evaluate phenotypic diversity in chile pepper fruits (Nankar et al., 2020; Pereira-Dias et al., 2020; Nimmakayala et al., 2021). The utility of the TA software to measure various fruit-related traits for large New Mexican pod-type chile pepper samples as well as the small fruit types, such as jalapeno, ornamental, and chiltepins, was demonstrated (Figure 2). Information collected from HTP can be combined with genetic marker and kinship information to predict traits of interest for GS (Dreisigacker et al., 2021). The fruit trait data can be used to improve accuracy for yield in chile pepper, by incorporating these as covariates in prediction models, as these traits are highly correlated and generally have higher heritability than yield (Naegele et al., 2016). Improvement of GS accuracy has been demonstrated by integrating secondary correlated traits, such as spectral reflectance indices for predicting grain yield in winter wheat (Sun et al., 2017; Crain et al., 2018; Juliana et al., 2019; Lozada and Carter, 2019), primarily a result of using genetically correlated traits for GS (Jia and Jannink, 2012). The World Vegetable Center has recently utilized a field-based automated HTP system to evaluate 300 members of a *Capsicum* core collection during both hot and optimal seasons. Data on more than 75 different traits using both manual recordings of yield and yield component traits and multispectral imaging of physiological and morphological traits including leaf angle, pollen concentration and activity, and Normalized Difference Vegetative Index (NDVI) were collected and will be used for genomics and phenomics-assisted breeding (Barchenger and Lefebvre, 2021).

“Speed Breeding” is another approach that shows great potential in accelerating genetic gain (Li et al., 2018a; Hickey et al., 2019). This method involved growing plants under continuous light (20–22 h.) permitting plants to photosynthesize longer, resulting in faster growth and multiple generations per year, thereby allowing researchers to develop new varieties quicker (Watson et al., 2018; Bhatta et al., 2021). Speed breeding is implemented using growth chambers and glasshouses, with some modifications based on the crop species (Ghosh et al., 2018). The success of speed breeding for rapid generation advancement has been demonstrated in barley (*Hordeum vulgare*; Zheng et al., 2013), durum wheat (*Triticum durum*; Alahmad et al., 2018), spring wheat (Watson et al., 2019), oats (*Avena sativa* L.; González-Barrios et al., 2021), and canola (*Brassica napus* L.; Yao et al., 2016), among others. To date, there is no known report on the implementation of the speed breeding approach in chile pepper, although initial evaluation indicated that the speed breeding growing conditions for the day-neutral cowpea (*Vigna unguiculata* [L.] Walp.; Ehlers and Hall, 1996) can be adapted. Preliminary speed breeding experiments in chile pepper should target the “late maturing” and “low germination” species, such as the *C. chinense* and the *Capsicum annuum* var. *glabriusculum* (chiltepins), to accelerate their developmental phases.

## “Agricultural Robotics” and Mechanization of Chile Pepper

Robots and autonomous systems are currently being integrated in agricultural systems to address challenges, such as labor-intensive tasks (e.g., crop harvesting, mechanical weeding, thinning, and pesticide spraying), labor shortage, and increasing need to monitor crop health and environments for improved production (Bac et al., 2014; Bechar and Vigneault, 2016; Roldán et al., 2018). Despite the use of advanced technologies in other farming systems and greenhouses (Bonadies et al., 2016; Ruiz-Larrea et al., 2016), chile pepper is still managed mainly using conventional practices. The use of robotic systems that can replicate “human-like” harvesting could be a potential solution to propel mechanization of chile pepper, in addition to breeding and selection for machine-harvestable cultivars. Moreover, using a mobile network of sensors (e.g., unmanned ground and aerial robots) to monitor and collect data in an extended spatiotemporal manner would significantly help determine soil stress and manage water usage and soil condition.

**Figure 3:**
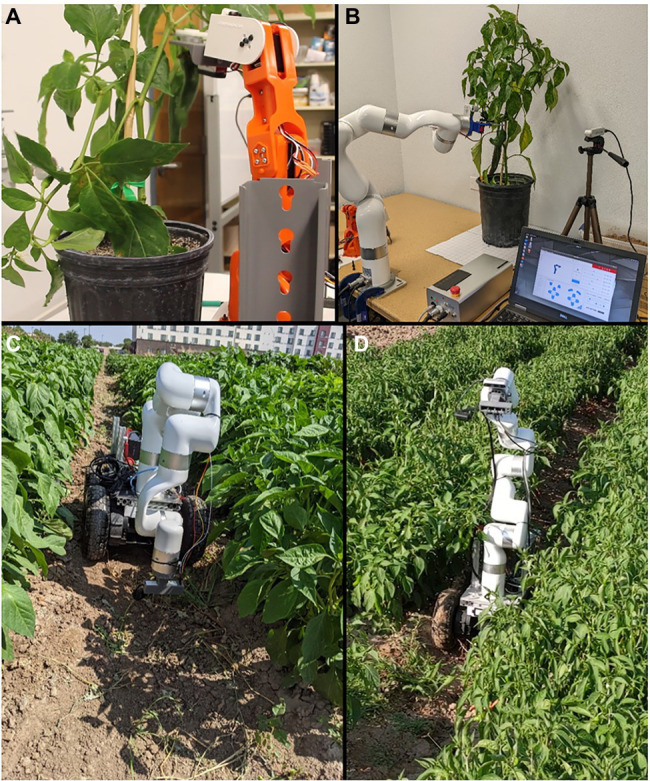
**(A)** A 5 degrees of freedom (DoF) robotic arm with a cutter end-effector harvesting green chile peppers in an indoor setting. **(B)** A 6 DoF robotic arm with integrated wrist camera and a cutter end-effector identifies the location of the stem and harvest green chile pepper. **(C)** Robotic soil moisture and temperature measurements at the NMSU’s Jose Fernandez Heritage Farm, Las Cruces, NM. **(D)** Image capturing and visual remote sensing using an autonomous ground mobile robotic arm in chile pepper testing field at the Leyendecker Plant Science Research Center, NMSU, Las Cruces, NM.

At NMSU, a series of feasibility studies on the use of robotic systems for chile pepper farming are being implemented. For example, in a preliminary work on the robotic harvesting for chile pepper, a 5 degrees of freedom (DoF; Figure 3A) and a 6 DoF (Figure 3B) robotic arms with a customized cutter end-effector have been investigated in a laboratory setting (Masood and Haghshenas-Jaryani, 2021). The harvesting robot showed promising results with high localization, detachment, and harvest success rates, low damage rate, and a cycle time comparable to the performance of other harvesting robots and human harvesters. The overall results have demonstrated the feasibility of using robotics approaches in harvesting chile pepper.

In another work, a robotic arm with sensors attached to a ground rover robot have been used for performing direct soil moisture-temperature measurements (Figure 3C) and remote visual sensing (image capturing using an RGB + D camera; Figure 3D) in chile pepper farms. The moisture sensor probe is inserted into the soil at multiple points along the rows of the chile pepper for measuring moisture level and temperature. Additionally, the camera attached to the wrist of the robotic arm takes RGB and infrared images from the plant and local soil for the visual analysis of the plant and soil’s health and physiological conditions through AI-enabled machine algorithms. Preliminary observations showed the exciting potential of the robotic system for collecting environmental data with a higher resolution for the spatial and temporal aspects of the targeted areas for determination of soil and plant stresses. The advantages of using ground robots over aerial systems include longer operation time, higher payload for carrying a variety of sensors for multimodal sensing, and less dependency to weather conditions. The multimodal data will used to map the environment and crops’ heterogeneous conditions that could be used for data-driven precision agriculture in chile pepper.

Mechanization of chile pepper harvesting is relevant for sustaining production amidst decreasing acreages resulting from labor shortages and increased labor costs. In recent years, the shortage of labor and prohibitive costs associated with manual labor have resulted in difficulty of harvesting and postharvest processing, consequently affecting the overall chile pepper production. This indicates a strong need to shift from the labor-intensive manual harvest to the mechanical harvesting of chile pepper. Growers have reported success using a tomato harvester that cuts the plants off at the grounds, then shakes the chile pepper fruit off the plants; however, the inclined double helix picking head continues to be the preferred machine for mechanically harvest of specialty peppers. While mechanical harvesting is commonly used for red chile, the New Mexican type green chile pepper is less amenable to machine-driven harvest due to the difficulty in removing the fruit, the potential of fruit damage, and the need for pedicel removal for a large segment of the industry.

A breakthrough in the effort to develop mechanically harvestable chile peppers is the recent release of “NuMex Odyssey,” a New Mexican pod-type green chile pepper amenable to machine-driven harvest (Walker et al., 2021). “NuMex Odyssey” has the ideal plant architecture for mechanical harvesting, such as a high percentage of plants with single stems and increased main bifurcation, using an inclined double-helix-type picking mechanism (e.g., the Yung-Etgar Moses 1010 machine harvester). Currently, the genetic basis of plant morphology and fruit destemming in New Mexican chile peppers is being examined using genomics-assisted breeding approaches, such as GWAS and QTL analysis, to gain a better understanding of the genetic architecture of these traits. In addition, “NuMex Odyssey” is utilized as a parental line for hybridization with other New Mexican pod-type chile peppers at the NMSU Chile Pepper Breeding Program to develop additional cultivars that are amenable to machine-driven harvest. Altogether, the development of machine-harvestable chile peppers would require a “systems approach,” integrating traditional and genomics breeding, the use of efficient harvesters, and relevant management practices (Funk et al., 2011).

Selection and breeding for lines with ideal plant architecture (ideotype) is important for the successful implementation of mechanical harvesting. Machine harvesting of fresh green chile peppers is impeded by the difficulty in destemming and the fruit damage associated with harvesting (Funk and Marshall, 2012). Plant architecture and growth habit, likewise, have a substantial impact on the efficiency of mechanical harvesting (Wall et al., 2003). Different types of machine harvesters need to be evaluated, as it might be essential to “fine-tune” these machines when harvesting different pepper types (New Mexican vs. jalapeño vs. cayenne, etc.). Furthermore, while the current production practice for “NuMex Odyssey” is through direct seeding in the field, as this results in taller and more upright plants for machine harvest (Walker et al., 2021), it will be necessary to examine the mechanical harvest potential of transplanted plants in the future. A strong collaboration between plant breeders, agro-mechanical engineers, and horticulturists should succeed in developing chile pepper cultivars that are amenable to machine harvesting in New Mexico and other growing regions of the world. Ultimately, the overall higher costs of chile pepper production in the United States compared to other countries would remain a potential drawback, even with the current shift to mechanize the chile peppers. Hence, it is also necessary to improve traits, such as nutritional quality and flavor to enhance consumer acceptance and product value for this important commodity.

## The “Panomics” Platform: Integrating Multi-Omics Tools for Trait Dissection in *CAPSICUM*

In addition to genomics-assisted breeding, transcriptomic, metabolomic, proteomic, and epigenomic profiling can be utilized to dissect the genetic basis of various traits in chile pepper. This integrated system, termed as “Panomics,” is expected to accelerate plant improvement through the discovery of target genes and biological pathways that are controlled by complex genetic and epigenetic mechanisms for “precision breeding” to develop elite lines (Weckwerth et al., 2020). Altogether, integrating various omics tools can render a better and deeper understanding of the genetic architecture of complex traits which consequently could drive genetic improvement in chile pepper. An integrative analysis of the transcriptome and proteome of two pepper varieties revealed a temporal specificity of key protein expression during fruit development (Liu et al., 2019). Metabolomic and transcriptomic analysis of two habanero (*C. chinense*) cultivars revealed variation in cutin composition and the upregulation of genes involved in cutin biosynthesis (Natarajan et al., 2020). Differentially expressed transcripts and metabolites were further observed in heat-tolerant and heat-sensitive pepper cultivars under heat stress (HS), where common HS-responsive genes were expressed in both genotypes (Wang et al., 2019). Combined metabolomic and transcriptomic analyses in chile pepper demonstrated the induction of jasmonic acid (JA) in response to mite infection and indicated the association of endogenous JA in conferring a strong defense mechanism during plant–arthropod interactions (Zhang et al., 2020).

Transcriptomic analyses of the cracking-susceptible pepper cultivar “L92” revealed differentially expressed genes during the fruit cracking process, where 45 genes were observed to be enriched in pathways related to cell wall metabolism and biosynthesis of lignin (Liu et al., 2022). Analyses of chile pepper transcriptome further demonstrated variation in the gene expression profiles during fruit development, particularly for genes related to cell cycle and division among wild and cultivated species (Martínez et al., 2021). RNA-sequencing (RNA-seq) previously identified genes, namely, *CA00g9220* and *CA00g96010*, to be strongly expressed in the resistant landrace, CM-334, upon infection by the pathogen, providing valuable information on the defense mechanisms of chile pepper against infection by *P. capsici* (Kim et al., 2019). In another study, at least 50 differentially expressed genes were identified through an RNA-seq approach, where the level of resistance and susceptibility to *P. capsici* was related to the differences in gene expression levels and molecular variations in the resistance mechanisms (Rabuma et al., 2022). RNA-seq can help identify Function Associated Specific Trait (FAST) markers, that are more informative as compared to the more “neutral” genome-wide SNP loci to improve accuracy for trait prediction using GS, genetic effect estimation, and parental line selection (Fu et al., 2017).

Gas chromatography–mass spectrometry (GC–MS) analyses of stip, a physiological disorder in bell pod-type peppers, detected significant variation in 13 metabolites related to fruit maturation and senescence (Fulton et al., 2021). Metabolomic profiling of mirasol pepper fruit samples infected by *Candidatus* Phytoplasma trifolii showed decreased levels of fructose, glucose, and capsaicin, suggesting that infection can reduce the pungency and nutraceutical value of mirasol peppers (Velásquez-Valle et al., 2020). Stress-related metabolite accumulation was further observed using a large-scale metabolomic characterization of mature fruit samples from 26 pepper cultivars (Kim et al., 2020). Proteomic characterization of broad mite (*Polyphagotarsonemus latus*) infected pepper samples using tandem mass tag (TMT)-MS analyses demonstrated the upregulation of proteins involved in plant defense response and hormone signal transduction (Patavardhan et al., 2020). Conversely, proteins involved in plant defense and hormone signaling were downregulated in response to green peach aphid (*Myzus persicae* Sulzer) infection (Florencio-Ortiz et al., 2021). Analysis of apoloplast proteome in chile pepper under drought conditions revealed increased peroxidase and reduced catalase activity and upregulation of 43 protein species related to stress response (Jaswanthi et al., 2019).

There has been growing evidence on the potential involvement of epigenetic factors, such as DNA methylation, histone acetylation, and chromatin remodeling, specifically on conferring *P. capsici* resistance in chile pepper (Du et al., 2021; Lozada et al., 2021b, 2021c). Examining the chile pepper epigenome using various techniques, such as DNA methylation PCR-based bisulfite sequencing and chromatin immunoprecipitation assays (Cazaly et al., 2019; Li, 2021), could therefore be relevant in breeding and selection for *P. capsici* resistant lines. The ability to induce mutations in any part of the genome through clustered regularly interspaced short palindromic repeats (CRISPR)/CRISPR-association protein 9 (CRISPR-Cas9; Jinek et al., 2012) also shows great possibility in fast-tracking chile pepper breeding, particularly for improving disease resistance. For instance, it might be possible to induce mutations using the CRISPR-Cas9 technology in susceptible chile pepper to confer resistance to *P. capsici* and other major diseases. Recently, an enhance resistance to anthracnose caused by *Colletotrichum* spp. was observed following a single transcript CRISPR/Cas9 modification of *CaERF28*, a major susceptibility gene (Mishra et al., 2021). One of the key issues that needs to be addressed is the absence of a stable regeneration system for the *Capsicum*. Chile pepper is recalcitrant to *in vitro* production and only genotype-specific systems have been developed, and hence for CRISPR to be appropriate, there would need to be a tissue culture-free system. Ultimately, a proof-of-concept study on the applicability of CRISPR in chile pepper is warranted. A proposed method on the integration of the panomics approach with genome editing could result in the identification of the majority of phenotypic variation in complex traits for a robust precision breeding for genetic improvement (Yang et al., 2021).

## Conclusion and Future Prospects

Modern chile pepper genetic improvement requires the integration of novel omics tools in the breeding pipeline to facilitate the development of improved cultivars. Using genomics-assisted breeding by discovering QTL and significant marker–trait associations, as well as complementing GWAS with GS, HTP, transcriptomics, proteomics, and metabolomics, and other omics tools (i.e., panomics; Weckwerth et al., 2020; Shen et al., 2022) to accelerate generation advancement can facilitate a deeper understanding of the genetic basis of different traits in chile pepper while improving gains achieved through selection. This, consequently, could drive important breeding and selection decisions for genetic improvement in chile pepper breeding programs. DNA marker genotyping will not be seen as a bottleneck in chile pepper breeding given the availability of automated, cost-effective, and high-throughput marker systems (e.g., SNPs) derived from NGS-based platforms, such as GBS. Recent developments on long-read sequencing approaches to examine structural variation in the genome (De Coster et al., 2021) will allow the construction of the “pan-genome” (Tettelin et al., 2005) for *Capsicum* in the future. This, in turn, would render novel insights into the genetic architecture of complex traits that were not fully captured using single reference genomes of cultivated chile peppers.

Field data collection particularly for yield and yield component traits might cause an issue as majority of the existing chile pepper cultivars are manually harvested. The recent release of “NuMex Odyssey,” a chile pepper cultivar amenable to mechanical harvesting, could potentially mitigate this constraint. Disease resistance will remain an important objective in chile pepper breeding programs and as knowledge on gene expression, epigenomic, and transcriptomic profiles become more available, the genetic dissection of disease resistance genes will result in better breeding, screening, and management practices.

Chile pepper improvement in the era of the multi-omics entails establishing and maintaining collaborative partnerships between the public and private sectors. Without students trained from public breeding programs (e.g., land-grant universities), private programs will experience a decrease of intellectual capital; whereas, without the private sector, cultivars and products developed by public programs will have difficulty reaching growers (Brummer et al., 2011). Breeding goals and objectives will depend on the current needs of growers, producers, and the chile pepper industry. Training of the next generation of plant breeders with these multi-omics approaches would be crucial to gain the necessary technical expertise and experience, especially on handling the “big data” generated from these tools. The abovementioned approaches offer a great promise for genetic improvement, yet further time and research work should be implemented to tap and realize their full potential in the context of chile pepper breeding programs. Ultimately, the tools presented herein will continue to “define” chile pepper breeding and improvement in the years to come.

## Author Contributions

DL conceived and wrote the first draft of the manuscript. PB, DB, MH-J, SS, and SW edited the manuscript. All authors contributed to the article and approved the submitted version.

## Funding

The Agricultural Robotics research is supported by a grant (Award number 2021-67021-34203) from USDA-NIFA and a seed funding from the NMSU Center of Excellence in Sustainable Food and Agricultural Systems (CESFAS) program.

## Conflict of Interest

The authors declare that the research was conducted in the absence of any commercial or financial relationships that could be construed as a potential conflict of interest.

## Publisher’s Note

All claims expressed in this article are solely those of the authors and do not necessarily represent those of their affiliated organizations, or those of the publisher, the editors and the reviewers. Any product that may be evaluated in this article, or claim that may be made by its manufacturer, is not guaranteed or endorsed by the publisher.
